# Efficacy, Trainability, and Neuroplasticity of SMR vs. Alpha Rhythm Shooting Performance Neurofeedback Training

**DOI:** 10.3389/fnhum.2020.00094

**Published:** 2020-03-20

**Authors:** Anmin Gong, Wenya Nan, Erwei Yin, Changhao Jiang, Yunfa Fu

**Affiliations:** ^1^School of Information Engineering, Engineering University of Armed Police Force, Xi’an, China; ^2^Department of Psychology, College of Education, Shanghai Normal University, Shanghai, China; ^3^Tianjin Artificial Intelligence Innovation Center (TAIIC), National Institute of Defense Technology Innovation, Academy of Military Sciences China, Beijing, China; ^4^Key Laboratory of Sports Performance Evaluation and Technical Analysis, Capital University of Physical Education and Sports, Beijing, China; ^5^School of Automation and Information Engineering, Kunming University of Science and Technology, Kunming, China

**Keywords:** neurofeedback, shooting performance, motor sensory rhythm, resting EEG, trainability

## Abstract

Previous literature on shooting performance neurofeedback training (SP-NFT) to enhance performance usually focused on changes in behavioral indicators, but research on the physiological features of SP-NFT is lacking. To explore the effects of SP-NFT on trainability and neuroplasticity, we conducted a study in which 45 healthy participants were randomly divided into three groups: based on sensory-motor rhythm of C3, Cz and C4 (SMR group), based on alpha rhythm of T3 and T4 (Alpha group), and no NFT (control group). The training was performed for six sessions for 3 weeks. Before and after the SP-NFT, we evaluated changes in shooting performance and resting electroencephalography (EEG) frequency power, participant’s subjective task appraisal, neurofeedback trainability score, and EEG feature. Statistical analysis showed that the shooting performance of the participants in the SMR group improved significantly, the participants in the Alpha group decreased, and that of participants in the control group have no change. Meanwhile, the resting EEG power features of the two NFT groups changed specifically after training. The training process data showed that the training difficulty was significantly lower in the SMR group than in the Alpha group. Both NFT groups could improve the neurofeedback trainability scores and change the feedback features by means of their mind strategy. These results may provide evidence of trainability and neuroplasticity for SP-NFT, suggesting that the SP-NFT is effective in brain regulation and thus provide a potential method to improve shooting performance.

## HIGHLIGHTS

- The neurofeedback based on SMR may be more effective than that based on alpha rhythm in improving the non-expert shooter’s pistol shooting performance.
- SMR and Alpha NFT have trainability. Participants can increase their feedback features and neurofeedback trainability scores during NFT stage.
- SMR and Alpha neurofeedback exert some effects on the effects on trainability and neuroplasticity, and feedback training can make specific directional changes in resting EEG.

## Introduction

Shooting is a simple motor behavior that can be easily affected by mental states such as attention and emotion. The relationship between shooting performance and central nervous system features has raised interest in many scholars, and this relationship has been widely investigated for shooting athletes through electroencephalography (EEG; Hatfield et al., 1984; Del Percio et al., 2009; Bertollo et al., 2016). For instance, it has been reported that during the preparation process in shooting, shooting experts show continuously increased alpha rhythm (8–12 Hz) at the T3 electrode (Hatfield et al., 1984). Furthermore, during the preparation process, a steady increase of theta rhythm (4–7 Hz) power at the frontal midline is shown in shooting experts rather than novice shooters (Doppelmayr et al., 2008), and the event-related desynchronization (ERD) and event-related coherence (ERCoh) of alpha rhythm in shooting experts is significantly less than that of novice shooters (Del Percio et al., 2009, 2011). In addition, a decrease in alpha power of the occipital region is reported during successful trials compared to fail shooting trials (Loze et al., 2001). These results suggest that shooting performance is closely related to the shooter’s brain activity during shooting preparation.

It is unknown if it is naturally possible to enhance shooting performance by interventions that influence brain activity. Therefore, in the movement neuroscience, brain regulation for enhancing shooting performance using neurofeedback training (NFT) has become a new research focus. NFT converts EEG signals into sound or animation, which is easily understood by the participants, to help people understand their own physical status. Participants can selectively enhance or suppress neurophysiological signals of a specified frequency band through repeated training and effectively regulate their brain function.

Many studies have used NFT to improve the sports performance of athletes (Raymond et al., 2005; Faridnia et al., 2012; Strizhkova et al., 2012; Cheng et al., 2015a; Mikicin et al., 2015; Ring et al., 2015). For instance, Raymond et al. (2005) improved the dance performance of the college dance sports team by increasing the alpha/theta ratio of the Pz electrode. Strizhkova et al. (2012) improved the complex coordinated activities performance of gymnasts by increasing alpha of F1, F2, P3 and P4 electrodes. Faridnia et al. (2012) reduced the sport competition anxiety of swimmers by increasing SMR and low beta and decrease theta and a high beta of C3 and C4 electrodes. Ring et al. (2015) improved the golfers’ putting performance by reducing theta and high alpha power of the Fz electrode. Mikicin et al. (2015) reduced student-athletes’ attention-reaction by increasing beta1 and SMR and decrease theta and beta2 of C3 and C4 electrodes. Cheng et al. (2015a) improved golfers’ putting performance by increasing SMR of the Cz electrode.

In the literature, only two studies attempted to improve shooting performance by NFT (SP-NFT). One SP-NFT study utilized SMR (Sensorimotor Rhythm, 12–15 Hz EEG rhythm, usually collected from C3, Cz, and C4), beta and alpha rhythm mixed NFT protocol to improve shooting performance (Rostami et al., 2012). This approach was taken because a lot of previous studies have found that the increase in SMR is often accompanied by attention increases. The NFT based on SMR has been widely used in the treatment of attention deficit hyperactivity disorder (ADHD), and its activities are also found to be closely related to the optimization of the skilled action execution motor performance such as golf putting and dart throwing (Vernon et al., 2004; Cheng et al., 2015a,b). Therefore, the researchers expected to increase the participants’ attention by increasing SMR, so as to improve their shooting performance.

On the other hand, it has been demonstrated that there is an asymmetry between the left and right hemispheres and an increase of alpha rhythm in the left temporal region during rifle and archery shooting preparation (Hatfield et al., 1984; Salazar et al., 1990). Based on this phenomenon, the other SP-NFT study utilized NFT to enhance EEG low-frequency activity over the left temporal region (T3) and successfully improved archers shooting performance (Landers et al., 1991). The authors explained this NFT could improve the shooting performance by simulating brain activity during actual rifle and archery shooting preparation.

Although both studies used NFT to improve shooting performance, it is unknown which of the two methods is more effective. In addition, according to the current standards of neurofeedback experimental research, there are still some problems in these two studies. Mirifar et al. (2017) suggested that neurofeedback combining visual and auditory feedback may be more effective than visual or auditory feedback alone. However, the two kinds of SP-NFT mentioned above only use visual feedback. Additionally, in Landers’s study, participants were tested for feedback effects after only one feedback training session. Some scholars have suggested that successful neurofeedback regulation may require a minimum of three to four sessions, and initial improvements can only be seen within the first five to ten sessions (Konareva, 2005; Hammond, 2011). More importantly, both studies only analyzed the efficacy of NFT from a kinematic perspective and did not provide a detailed analysis of the effect of NFT on trainability and neuroplasticity (Enriquez-Geppert et al., 2014). That is, there was no indication of whether the EEG features of feedback can be actively modulated by the participants during the training process, i.e., trainability, or if this training can produce some lasting changes to the EEG rhythm of the brain, i.e., neuroplasticity.

In previous NFT studies, the NFT trainability was firstly examined before confirming that the NFT can affect cognition/behavior. For instance, Cho et al. (2007) confirmed the NFT trainability of alpha activity in the midline parietal region and found that NFT could enhance participants’ ability to maintain alpha activity. Zoefel et al. (2011) first studied the trainability of upper alpha NFT in the parietal and occipital regions and found the effect of this NFT on cognitive improvement. Enriquez-Geppert et al. (2014) demonstrated the trainability of frontal midline theta NFT and revealed the role of training in cognitive ability improvement. These literatures suggest that proving NFT trainability is the basis of studying the effects of NFT on cognition/behavior.

Studies regarding neuroplasticity are mainly based on the correlations between neurological characteristics and cognitive/behavioral indicators. Many studies found a significant correlation between neurological characteristics of resting states and behavioral/cognitive indicators (Babiloni et al., 2010; Zhou et al., 2012; Wan et al., 2014; Zhang et al., 2015). Therefore, scholars believe that if NFT changes not only the cognitive/behavioral indicators but also the relevant neurological characteristics, it will be an important evidence supporting its influence in cognition/behavior by altering the neurological characteristics. Many scholars have studied the neuroplasticity generated by NFT from different perspectives. Besides finding that NFT can produce neuroplasticity changes in resting EEG, NFT can lead to changes in white and gray matter in the brain and the resting brain network features (Cho et al., 2007; Zoefel et al., 2011; Ghaziri et al., 2013; Kluetsch et al., 2014).

Here we sought to improve SP-NFT by using both visual and auditory feedback and increasing the number of sessions, while also examining trainability and neuroplasticity. To examine trainability, we recorded the extent to which each participant could actively modulate EEG features during the training process. To examine neuroplasticity, we measured resting-state EEG before and after NFT. Current studies have found significant correlation between resting EEG features such as individual alpha frequency (IAF) and frequency band power and the performance of participants completing cognitive tasks, motor imagery tasks, and even shooting tasks (Koch et al., 2008; Zhou et al., 2012; Zhang et al., 2015; Gong et al., 2017). Other studies also found that there were significant differences in resting EEG features among participants of different sport levels (Gong et al., 2017). Therefore, we believe that resting EEG is a good neurological indicator reflecting the brain “baseline” state and can be used to analyze the brain neuroplasticity induced by training (Khanna et al., 2015; Zhang et al., 2015).

Previous studies on SP-NFT have not specifically focused on the changes in resting EEG caused by NFT. Therefore, they could not fully demonstrate whether NFT really changed brain activity or whether the observed effect was simply a placebo effect of neurofeedback (Schönenberg et al., 2017; Xiang et al., 2018). These research limitations are one of the main reasons why NFT technology has been controversial and has not gained as much popularity in the kinematics field (Gruzelier, 2014; Mirifar et al., 2017).

To better explore the effects of SP-NFT on trainability and neuroplasticity, we hypothesized the following: (1) SP-NFT has an effect on trainability, participants can master the SP-NFT, and improve neurofeedback trainability scores; and (2) SP-NFT has an effect on neuroplasticity, such that resting-state EEG activity is significantly changed.

## Materials and Methods

### Participants

The 45 healthy college students (male, age: 19.5 ± 2 years) from the Armed Police Engineering University voluntarily took part in this research. All participants completed pistol course learning, had qualifying grades, and mastered fixed-target pistol shooting skills. Before training, all participants were divided into three groups according to their age, height, weight, and somatotype. The SMR group (*N* = 15) aimed to enhance SMR (12–15 Hz) power of C3, Cz, and C4 channel, similar to the study of Rostami et al. (2012). The Alpha group (*N* = 15) training aimed to enhance the alpha rhythm (8–12 Hz) power of T3 channel and decrease that of T4 channel, similar to the study of Landers et al. (1991). The control group (*N* = 15) did not receive NFT, just underwent a shooting test. This study protocol was in accordance with the Declaration of Helsinki and approved by the university ethics committee. Before the experiment, the experimental processes and purpose were explained, and the written informed consent was obtained from all participants. Participants were free to withdraw from the experiment at any time.

### Experimental Design

**Figure 1:**
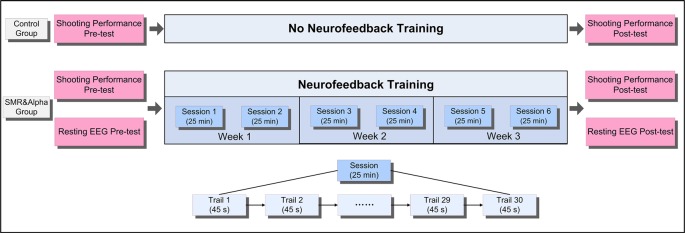
The experimental design and flow.

Figure 1 shows the experimental design and flow of our study. First, all participants underwent a shooting performance pre-test and a resting EEG pre-test within 3 days before NFT. Next, during the NFT period, the SMR group and Alpha group underwent six NFT sessions within 3 weeks at convenience. Finally, within 3 days after all NFTs, they completed a shooting performance post-test and a resting EEG post-test. It is worth noting that the control group only underwent shooting performance pre-test and post-test, and all participants did not perform any excess shooting tasks during the 3 weeks.

### Shooting Performance Pre-test and Post-test

All participants underwent two pistol shooting tasks to evaluate the effect of NFT on shooting performance. The shooting test was organized by the College of Basic Military Education. Participants used a type 92 pistol, aiming at a target 25 m away. The dimensions of the target were 52 × 52 cm. It included 10 rings, with a diameter of 10 cm and 10 ring edges, each extending 5 cm followed by 9, 8, 7, and 6 rings. The corresponding shooting score was obtained by hitting the position on the target, and a miss was recorded as 0. For example, if the shooter hit the center of the target, a score of 10 was recorded. Every shooter was asked to take a standing position in a single firing mode. Prior to each shot, the participants were informed of their previous shooting score. All participants performed 25 shots at their own pace.

### Acquisition and Preprocessing

The resting EEG acquisition device was a Beijing SymTom 32-D EEG amplifier. The EEG signals were recorded from 32 electrodes according to the international 10-20 system, including Fp1, Fp2, F3, F4, C3, C4, P3, P4, O1, O2, F7, F8, T3, T4, T5, T6, Fz, Cz, Pz, FC3, FC4, CP3, CP4, FT7, FT8, TP7, TP8, FCz, CPz, Oz, PO3, and PO4. The ground electrode was placed over the forehead and the reference was the left and right mastoids. The impedance of each electrode was kept below 5 kΩ and the sampling frequency was 1,000 Hz. The participants were asked to sit on soft and comfortable seats, while asking them to remain relaxed but not to fall asleep, and do not try to recall anything. EEG data of participants were collected with eyes-closed for 5 min and eyes-open for 5 min. To control the alertness level, the participants’ behavior and the quality of the EEG signals were monitored online in real-time. If the EEG had abnormal changes due to coughing, manual activity, sleepiness, etc., the participants were verbally reminded to cooperate.

The EEG signals at all recording channels were analyzed offline in MATLAB (2014a) and EEGLAB toolbox (Delorme and Makeig, 2004). The signals were firstly segmented into 2 s epochs and the data epochs corrupted by artifact were rejected by visual inspection (Delorme and Makeig, 2004). Independent component analysis (ICA; fast ICA algorithm) was used to remove blinking artifacts during the eyes-open resting state (Jung et al., 2001). Blinking artifacts were recognized by calculating the correlation coefficient with the EEG signals of Fp1 and Fp2 channels. Any component with a correlation coefficient greater than 0.8 was considered as the blinking artifact and was removed (the 0.8 was empirical data). Then a 6-order Butterworth band-pass filter of 0.5–40 Hz was used to filter the signal. Finally, the clean EEG data were used for subsequent frequency band power analyses. The frequency band power of the EEG signal was calculated using the Welch method. The calculated time window spanned 5,000 sampling points (5 s) and the overlap rate was 50%.

To control for differences in individual frequency bands between participants, the frequency bands of interest were defined relative to the IAF (Klimesch, 1999). The EEG power spectrum is calculated in the occipital region during the resting eyes-closed state, and the peak frequency position is found in the range of 7–13 Hz frequency bands. Finally, this peak frequency position was recorded as the IAF of the participants, and other frequency bands were defined relative to the IAF as follows: theta as IAF −6 Hz to IAF −3 Hz, alpha as IAF −2 Hz to IAF +2 Hz, beta as IAF +3 Hz to IAF +20 Hz.

### Implementation of Shooting Performance NFT

According to previous studies, an effective neurofeedback experiment should preferably include at least five training sessions, and the interval between two training sessions should be at least 1 day (Mirifar et al., 2017). Therefore, our study design included six sessions of SP-NFT in 3 weeks, in which each participant would undergo two sessions a week with an interval of at least 1 day in-between (Figure 1).

To ensure double-blinded NFT, experimenters were only required to enter the unique identity number of the participants into the NFT system (Ros et al., 2019). The system automatically identified the feedback type of the participants and ran the corresponding NFT mode. Therefore, the experimenter was not aware of the feedback mode in which the participant was training.

**Figure 2:**
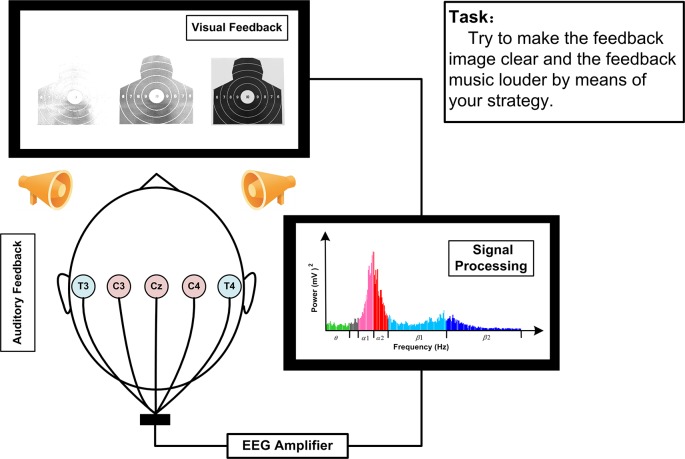
Schematic figure of NFT.

The EEG signals during SP-NFT were recorded from Cz, C3, C4, T3, and T4 for all participants in both NFT groups (Figure 2). The EEG signals were transmitted to the computer by the USB interface, and the SP-NFT program written in MATLAB 2014 was used to process and calculate the EEG features for feedback to participants in real-time. The feedback feature of the SMR group was the average SMR power of Cz, C3, and C4, while the feedback feature of the Alpha group was the alpha power of the T3 electrode minus alpha power of T4 electrode.

Each training session lasted about 25 min and included 30 trials. Each trial included a 15-s relaxation break and 30-s SP-NFT. During the relaxation break, the participants sat in silence and did not deliberately recall anything. At the end of the relaxation stage, the median value of the feedback feature in this period was shown as a red line on the feedback interface and served as the baseline of the SP-NFT trial. In the training stage, the feedback feature of the participants was calculated in real-time and presented to the participants in the form an image of a shooting target at different clarity levels, and a dynamic blue curve, both indicating the magnitude of the feedback feature. The participants were requested to focus on the shooting target image and to try to improve the feedback feature by means of their own mental strategy. Participants are advised not to be overly nervous during the NFT period, to remain relaxed but focused, to try to imagine the movements of the shooting preparation stage or to focus on the target image on the computer screen. When the feedback feature increases, the curve rises and the image becomes clearer; if the feedback feature decreases, the curve falls and the image becomes less clear. To implement the important auditory component of our NFT paradigm, the system included a digital sound equalizer to control the play of feedback music. The parameters of the equalizer were controlled according to the real-time feedback EEG feature: at the beginning of the training stage, the music was lowest, and when the magnitude of the feedback feature was higher than the baseline, the feedback music would gradually increase in volume and clarity.

To evaluate the success of SP-NFT, the system automatically calculated the neurofeedback trainability scores after each trial. The trainability score was determined as the ratio of time the feedback features were higher than the baseline over the total time of the training period. If the feedback features were always higher than the baseline during the feedback period, the neurofeedback trainability scores were recorded as 100 points. On the contrary, if the feedback features were always lower than the baseline, the neurofeedback trainability scores were recorded as 0. At the end of each feedback trial, the feedback score displayed and shown to the participant.

### Subjective Task Appraisal of Participants

To compare the subjective outcomes of SP-NFT between SMR groups and the Alpha group, a participants’ subjective task appraisal scale was used to evaluate the two kinds of training. After each SP-NFT session, participants reported the degree of fatigue, commitment, and difficulty in relation to the training. The reported scale used a five-level Likert scale: one indicated the lowest degree and five indicated the highest degree. For example: for fatigue degree, one means no fatigue at all, and five means very tired.

### Statistical Analysis

It is noteworthy that we tested the probability distribution of the samples using the Kolmogorov–Smirnov test. Regarding shooting performance, resting EEG IAF, and resting EEG frequency band power, it was found that these samples were not all subjected to Gaussian distribution. Thus, we used the Wilcoxon signed-rank test for these samples to examine the difference between pre-test and post-test in each group. For subjective task appraisal index and NFT trainability score, feedback feature and repeated-measures ANOVA was applied because all the samples were subjected to Gaussian distribution.

#### Shooting Performance Index

For each participant, the shooting performance index was the mean value of the 25 shot scores. To examine the differences in shooting performance between pre-test and post-test, Wilcoxon signed-rank test was used to test the median difference of shooting performance index between pre-test and post-test in the SMR group, Alpha group, and control group, respectively. Then, to compare the difference in the effect of the SMR group and the Alpha group on the shooting performance, we also carried out the Wilcoxon rank-sum test on the shooting scores of the post-test minus pre-test for the SMR group and the Alpha group.

#### Subjective Task Appraisal Index

For the subjective task appraisal index of the two SP-NFT groups, a statistical analysis was applied to test the difference between the two groups. To test whether the participants had experienced any changes during the six training sessions based on the subjective task appraisal, a repeated-measure ANOVA with the factors group (SMR vs. Alpha) and the factors session was performed (1–6).

#### Neurofeedback Trainability Scores and Feedback Feature

To examine the neurofeedback trainability of two SP-NFT groups, two repeated-measures ANOVAs were performed. The first ANOVA with factors group (SMR vs. Alpha) and factors session (1–6) compared the real-time calculated neurofeedback trainability scores for each trial, averaged within each session and compared across sessions and groups. This score was calculated as the ratio of the time that the feedback feature remained above baseline for each NFT trial. The second ANOVA with within-subject factor state (Train vs. Relax) and within-subject factor session (1–6) was conducted for the averaged values of the individualized feedback feature across all trials within each session.

#### Resting EEG Rhythm Power Indexes

Finally, we analyzed the changes in resting EEG features before and after training from three perspectives: (1) the resting EEG IAF (calculated by the eyes-closed resting EEG in the occipital region); (2) changes in the EEG power spectrum and the specific frequency band power in channels of interest (COI) during training; and (3) calculation of the average whole-brain EEG frequency band power topographic map of all participants and visually comparing the changes of the whole brain EEG before and after training. For the resting EEG IAF and band power of COI, the difference between pre-test and post-test was tested using the Wilcoxon signed-rank test. The visual comparison of the whole brain map only reflects the change of the average power spectrum before and after the feedback training; it does not reflect statistical testing. All statistical tests are performed in MATLAB 2014.

## Results

### Shooting Performance Before and After the SP-NFT

**Figure 3:**
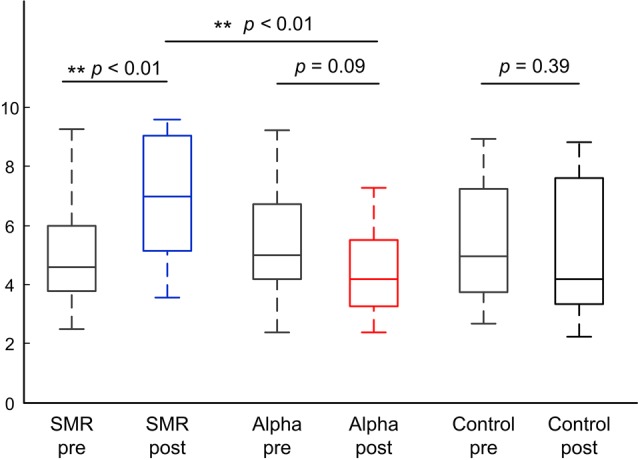
The shooting scores box plot of the pre-test and post-test of three groups.

Figure 3 indicates the comparisons of the shooting scores of the three groups in pre-test and post-test. The left is the SMR group, the middle is the Alpha group, and the right is the Control group. Blue, red, and black box indicates post-test shooting performance and the gray box indicates pre-test shooting performance. The statistical results show that the median shooting score after training is significantly higher than that before training in the SMR group (*z* = −3.55, *p* < 0.01). The median shooting score after training is marginal significantly lower than that before training in the Alpha group (*z* = 1.80, *p* = 0.09). The median shooting score have no significant change in control group (*z* = 0.85, *p* = 0.39). In terms of shooting score difference between two SP-NFT groups, the SMR group is significantly higher than the Alpha group (*z* = −3.06, *p* < 0.01).

### Subjective Task Appraisal of Participants

Table 1 shows the mean and SD of the three subjective task appraisal indexes for the six feedback sessions. As can be seen from the table, the fatigue degree of the two groups was approximately 2, while the degrees of effort and difficulty were approximately 3 in both groups. Results of repeated-measure ANVOA statistical analyses show that for fatigue index, the group factor effect is not significant *F*_(1,14)_ = 0.44, *p* > 0.05, and the session factor effect is not significant *F*_(5,70)_ = 0.47, *p* > 0.05. For commitment index, the group factor effect is significant *F*_(1,14)_ = 10.54, *p* < 0.01, such that the Alpha group is significant higher than SMR group; the session factor effect is not significant *F*_(5,70)_ = 0.68, *p* > 0.05. For difficulty index, the group factor effect is significant *F*_(1,14)_ = 17.02, *p* < 0.01, with higher difficulty in the Alpha group compared to the SMR group; the session factor effect is not significant *F*_(5,70)_ = 0.18, *p* > 0.05. The interactions between session and group of the three indicators are not significant.

**Table 1:** Mean and SD of each feedback subjective task appraisal of the two neurofeedback training (NFT) groups.

	Session 1	Session 2	Session 3	Session 4	Session 5	Session 6
Fatigue (SMR group)	2.27 (1.03)	1.93 (0.70)	2.20 (0.86)	2.20 (0.94)	1.93 (0.96)	2.00 (1.07)
Fatigue (Alpha group)	2.13 (0.74)	2.13 (0.92)	2.00 (0.85)	1.93 (0.80)	2.00 (0.85)	1.80 (0.68)
Commitment (SMR group)	2.80 (0.56)	2.93 (0.70)	2.80 (0.86)	2.53 (0.92)	2.87 (0.99)	2.60 (0.74)
Commitment (Alpha group)	3.40 (0.99)	3.13 (0.92)	3.27 (0.80)	3.00 (0.76)	3.00 (0.93)	3.13 (0.74)
Difficulty (SMR group)	2.27 (1.03)	2.33 (0.90)	2.33 (1.11)	2.33 (1.11)	2.40 (1.12)	2.40 (1.06)
Difficulty (Alpha group)	2.93 (0.59)	2.67 (0.82)	3.00 (0.65)	3.07 (0.80)	2.87 (0.83)	2.93 (0.96)

*From top to bottom, there are the results of fatigue, commitment, and difficulty of SMR group and Alpha group, respectively, and from left to right, session 1 to session 6*.

### Dynamic Changes in Parameters of SP-NFT

#### The Dynamics of Trainability Scores

**Figure 4:**
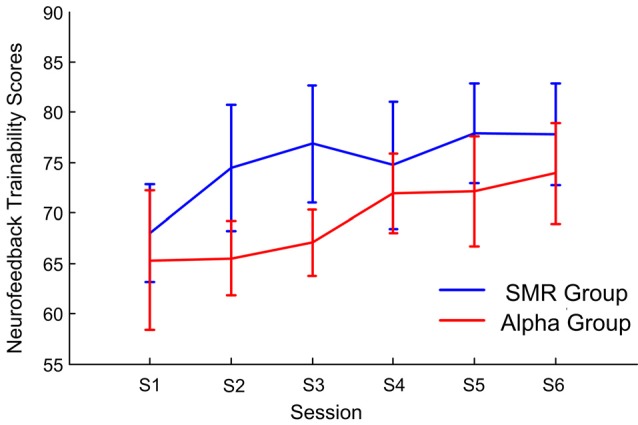
The average and 1.96× standard errors of the neurofeedback trainability scores vary with the feedback session.

Figure 4 shows the mean and 1.96× standard errors of neurofeedback trainability scores for each session. The horizontal axis is the feedback training session, the vertical axis is the neurofeedback trainability scores, the blue line indicates the SMR group, and the red indicates the Alpha group. The neurofeedback trainability scores of both NFT groups increased as the number of training sessions increased. The results of repeated—measure ANOVA showed the group factor effect is significant (*F*_(1,14)_ = 5.43, *p* < 0.01, *post hoc*: SMR group > Alpha group). For the session factor effect is significant (*F*_(5,70)_ = 3.13, *p* < 0.01, *post hoc*: session 6 > session 1). This finding indicated that participants could master the neurofeedback well after six sessions of SP-NFT, and the neurofeedback trainability scores were significantly improved.

#### The Dynamic of Feedback Features

**Figure 5:**
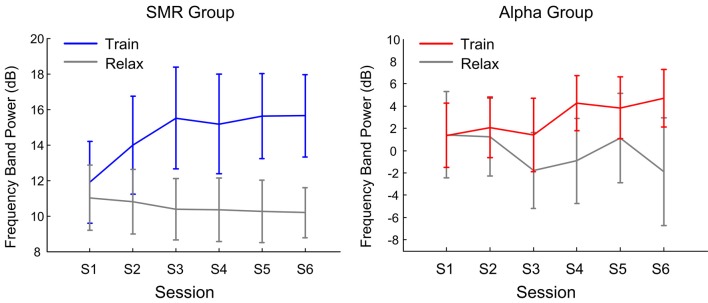
The average and 1.96× standard errors of the feedback feature varies with the feedback session.

Figure 5 shows the dynamics of feedback features with training sessions. The left side is the SMR group and the right side is the Alpha group. The gray line is the feedback feature in a resting state; the blue and red lines indicate the feedback feature of SMR and Alpha groups, respectively. For the SMR group, the results of repeated—measure ANOVA showed that the state factor effect was significant (*F*_(1,14)_ = 78.25, *p* < 0.001, *post hoc*: Train > Relax) while the session factor effect was not significant (*F*_(5,70)_ = 0.68, *p* > 0.05). For the Alpha group, the results of repeated- measure ANOVA showed that the state factor effect was significant (*F*_(1,14)_ = 7.32, *p* < 0.01, *post hoc*: Train > Relax) whereas the session factor effect was not significant (*F*_(5,70)_ = 0.28, *p* > 0.05). Comparing the two groups, it was found that the feedback features of both NFT groups in the training state were significantly higher than that of the relaxation state, while the *F* value of the Alpha group was lower than that of the SMR group. This may also be one of the reasons why participants in the Alpha group considered the training was more difficult than those in the SMR group.

### The Effect of SP-NFT on the Resting EEG

#### Comparison of Pre-test and Post-test IAF

The median value (SD) of IAF calculated according to the resting eyes-closed EEG is as follows. In the SMR group, the pre-test is 10.64 ± 0.69 Hz and the post-test is 10.45 ± 0.56 Hz, and there was no significant difference between pre-test and post-test (*z* = 0.99, *p* = 0.32). In the Alpha group, the pre-test is 10.30 ± 0.83 Hz and the post-test is 10.23 ± 0.92 Hz, and there was no significant difference between pre-test and post-test (*z* = 1.42, *p* = 0.16).

#### Comparison of Pre-test and Post-test Resting EEG Power Spectrum on COI

**Figure 6:**
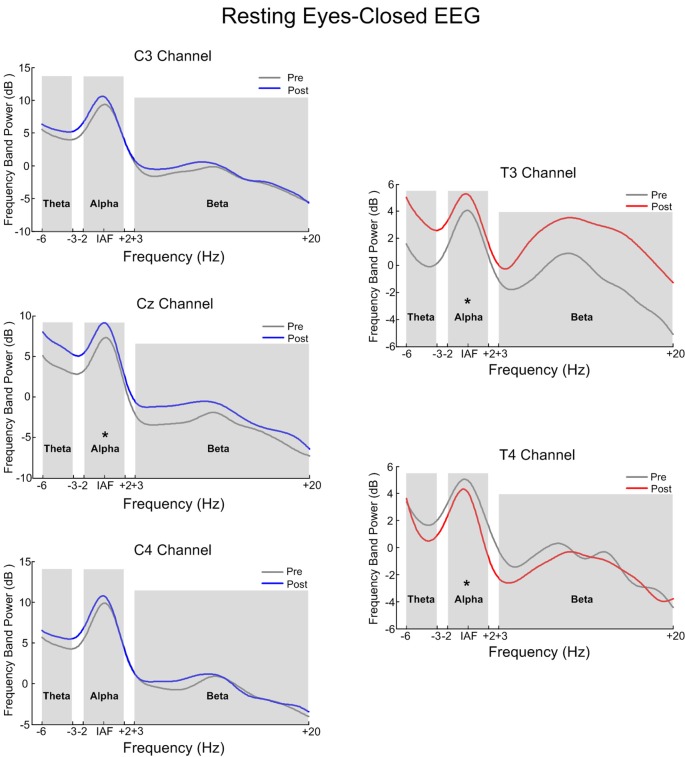
The contrast of pre-test and post-test electroencephalography (EEG) power spectrum of the eyes-closed resting state. *Indicates there was significant difference in the frequency band power between the pre-test and post-test (*p* < 0.05).

**Figure 7:**
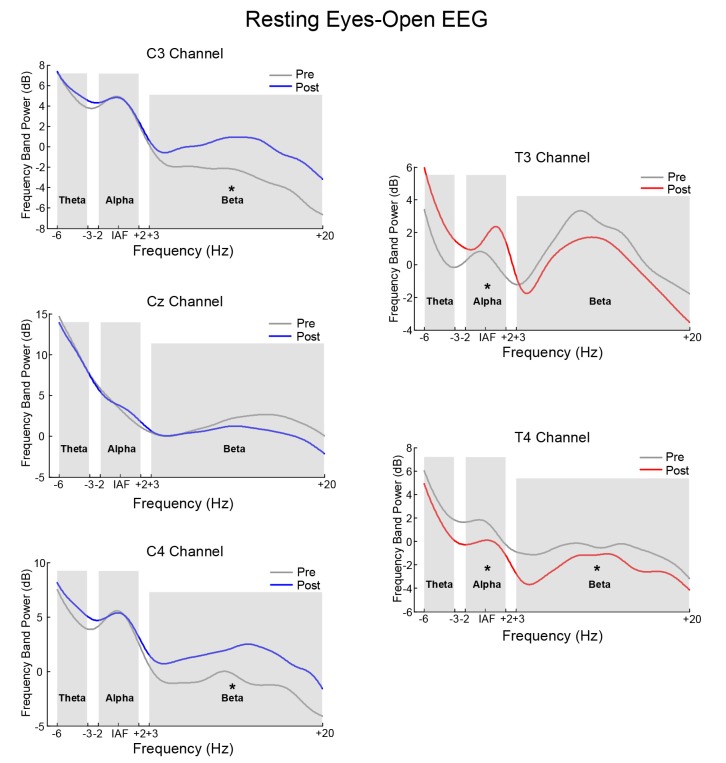
The contrast of pre-test and post-test EEG power spectrum of the eyes-open resting state. *Indicates there was significant difference in the frequency band power between the pre-test and post-test (*p* < 0.05).

Figure 6 is the power spectrum of the resting eyes-closed EEG on the feedback channel before and after training for the SMR group and Alpha group, respectively. The left side corresponds to the SMR group and the right side, to the Alpha group. The asterisk (*) indicates that there was a significant change in the frequency band power between before and after training (*p* < 0.05). Figure 7 shows the power spectrum of the resting eyes-open EEG, with similar details as in Figure 6. The power spectrum of the two groups changed after the training compared with before the training, and the change was in the same direction as the direction of SP-NFT: the training for the SMR group consisted in increasing the SMR power of Cz, C3, and C4, causing the resting eyes-closed alpha frequency power of the Cz channel (*z* = −2.96, *p* < 0.05) and the resting eyes-open beta frequency power of the C3 and C4 channels to significantly increase (C3: *z* = −4.39, *p* < 0.05; C4: *z* = −3.22, *p* < 0.05). The training of the Alpha group consisted of increasing the alpha power of the T3 electrode and decreasing the alpha power of the T4 electrode, leading to a significant increase of the resting eyes-closed alpha frequency power at the T3 electrode (*z* = −3.01, *p* < 0.05), significant decreases of the resting eyes-closed and eyes-open alpha frequency power (eyes-closed: *z* = 2.86, *p* < 0.05; eyes-open: *z* = 2.88, *p* < 0.05) as well as the resting eyes-open beta frequency power at the T4 electrode (*z* = 2.95, *p* < 0.05).

#### Comparison of the Whole Brain Resting EEG Frequency Band Power

**Figure 8:**
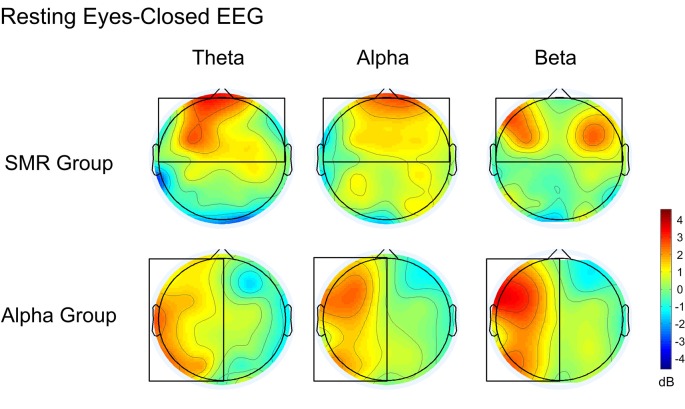
The difference of EEG power between pre-test and post-test of the eyes-closed resting state.

**Figure 9:**
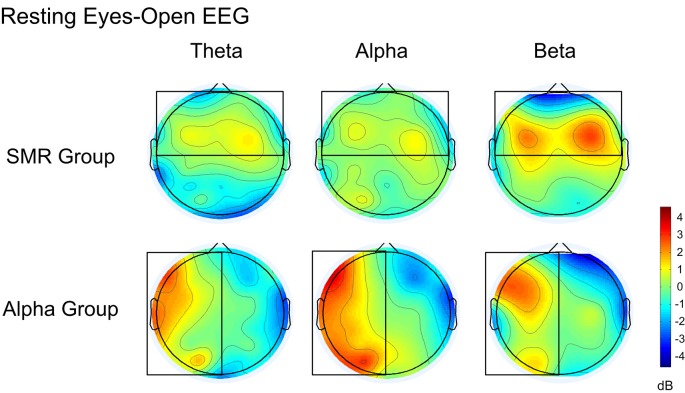
The difference of EEG power between pre-test and post-test of eyes-open resting.

Figure 8 is a whole-brain topographic map of the resting eyes-closed EEG frequency power difference between the NFT groups before and after training. The upper is the SMR group and the lower is the Alpha group. From left to right are theta, alpha and beta frequency band. Red indicates that the EEG power of the post-test is higher than that of the pre-test, and the blue indicates that the EEG power of the post-test is lower than that of a pre-test. Figure 9 is a whole-brain topographic map of the resting eyes-open EEG frequency power difference between the groups before and after training. In the SMR group, three frequency bands in the prefrontal, frontal channels, and central channels were increased after feedback. For the Alpha group, the frequency band power of the left hemisphere increased after feedback, while that in the right hemisphere was slightly weakened. The resting EEG changes in both NFT groups were consistent with the enhanced direction of SP-NFT.

## Discussion

In this article, we explored and compared the efficacy, trainability, and neuroplasticity of SMR vs. alpha rhythm SP-NFT. We improved traditional neurofeedback paradigms by including an auditory feedback component, in addition to a visual feedback component. Furthermore, we increased the number of NFT sessions and carried out six NFT sessions for each participant, rather than the typical one or two sessions. In addition, we have also taken into account trainability and neuroplasticity, which have not been fully explored by previous researchers.

### The Effect Analysis of SP-NFT on Shooting Performance

We evaluated the shooting performance of the three groups after SP-NFT and found that the groups achieved different results. The median shooting score reflects the overall shooting level. There was a significant improvement in the shooting performance of the SMR group, whereas there was a decrease in the performance of the Alpha group. Significant improvement in the shooting performance of the participants who participated in SMR training is consistent with the results of Rostami et al. (2012). In previous findings, an increase in SMR was often accompanied by an increase in attention (Vernon et al., 2004; Cheng et al., 2015b). NFT based on SMR has been widely used in the treatment of ADHD, and its activities have been found to be closely related to the optimization of the skilled action execution motor performance, such as golf putting and dart throwing (Vernon et al., 2004; Cheng et al., 2015a,b). These results also extend the potential facilitation effects of SMR training to athletes and healthy people in need.

Nonetheless, participants who underwent alpha training did not achieve improvement and even displayed a decline in shooting performance. The feedback feature of the Alpha group was characterized by the alpha power difference between T3 and T4, and the participants were given positive feedback when the feedback feature increased. That is, the participants got positive feedback irrespective of the increase in alpha power of T3 or a decrease in the alpha power of T4. Collura (2013) pointed out that in neurofeedback experiments, the training aimed at reducing activity may be a kind of “squeeze” enhancement training. Enhancing or reducing EEG activity in a brain region may lead to increased activity in that region (Plotkin and Rice, 1981). Although researchers try to suppress the activity of a certain brain area, the results of the training may instead lead to the enhancement of the brain area. According to this view, Alpha group enhanced the alpha rhythm of left temporal region and decreased the alpha rhythm of the right temporal region; however, the results may strengthen the activity of both temporal hemispheres of the participants because the participants did not acquire their shooting skills through training and the shooting performance did not improve. Therefore, we speculate that if the feedback feature is changed to the alpha power of the Hemi-temporal region, better training results may be obtained.

On the other hand, in terms of EEG features, Landers et al. (1991) used the Slow Cortical Potential (SCP) signal, whereas we used the alpha rhythm signal in the present study. Although the experimental principle is the same, it may lead to different experimental results. In terms of participant selection, the participants of Landers et al. (1991) were pre-professional athletes, while our participants were military students with amateur shooting levels. These differences may also lead to the inconsistency between the results of our research and previous studies.

### The Effect of SP-NFT on Trainability

We defined trainability as the ability of participants to control their own NFT features in training. The experimental results in “Dynamic Changes in Parameters of SP-NFT” section show that the trainability in both groups increased gradually throughout the six SP-NFT sessions. Neurofeedback trainability scores for the sixth session were significantly higher than those for the first session. The feedback features increased gradually up to the first four sessions, and the change range between the fifth and sixth sessions was stable. These results are consistent with previous studies of alpha training and frontal middle line theta training (Cho et al., 2007; Zoefel et al., 2011; Enriquez-Geppert et al., 2014). In addition, we also inquired about the strategy used by the participants who had high neurofeedback trainability scores during NFT. Most of them reported that they were “focusing on one point in the target image” or “to concentrate on the motor imagination of the shooting preparation stage.” These results indicate that both feedback training modes are effective and the participants can actively modulate their EEG rhythm power features. In particular, the subjective task appraisal showed that the degree of difficulty for the Alpha group was significantly higher than for the SMR group, which indicates that SMR feedback might be easier and more convenient for participants.

### Effects of SP-NFT on Neuroplasticity on Resting EEG

The third focus of our research was to study the effects of SP-NFT on neuroplasticity, that is, whether SP-NFT can change the brain neural activity and whether the brain activity of the participant has changed throughout a period of training (Ghaziri et al., 2013; Megumi et al., 2015; Faller et al., 2019). In this study, we tested this by examining the resting EEG of the participants before and after NFT.

The results of our study showed that after training, the resting eyes-closed and eyes-open EEGs significantly changed in both NFT groups. From the power spectrum of COI and the power topographic map of the whole brain, the position at which the resting state EEG changed was the region where the feedback electrode was placed. This suggests that NFT can cause specific changes in the channels and frequency bands involved in feedback, and it provides evidence that NFT exhibits neuroplasticity at the EEG level. However, from the brain topographic map, we find that in addition to the trained target channels and frequency bands, the adjacent channels and frequency bands have also undergone trend changes, which are a non-specific change. Collura (2013) suggest this could be a kind of influence of the “entrainment” effect: in addition to the frequency bands and channels involved in feedback, the EEG power of other nearby frequency bands and the cerebral cortex also showed a certain degree of change. Other neurofeedback also has reported similar phenomena (Cheng et al., 2015a).

The resting-state EEG of the participants changed significantly after SP-NFT, which is not only strong evidence that neurofeedback promotes neuroplasticity, but it also may provide a reason why SP-NFT can improve behavior indices (shooting performance). This shows that in future training processes of skill learning, we may be able to incorporate appropriate NFT throughout the entire training process and improve the participants’ attention and mental abilities, thereby improving the participants’ physical ability and skill level.

### Research Limitations and Improvements

Through comparative experiments, we studied the effects of two kinds of SP-NFT on training ability and brain regulation. However, based on the experimental conditions, the study had several limitations and could have benefited from some improvements:

(1) No active control group was set up. In this study, we divided the participants into two training groups and one passive control group. The control group only underwent shooting performance pre-test and post-test. Although such experimental settings have little effect on the research of trainability and neuroplasticity, there may be a placebo effect in the study of behavioral indicators. The control group did not undergo NFT, thus the observed effects may not be due to specificity of training in the given EEG frequency bands. In later studies, an active control group should be set up to avoid this problem.
(2) We used a single feedback interface. Although we have adopted the feedback mode of combined stimuli of sound and image, and further optimized the traditional feedback system, the same feedback images and music were applied in all six SP-NFT sessions, which may cause the participants to lose interest in the later stages of training, and thus, lead to a decline in the effect of SP-NFT.
(3) In the alpha rhythm NFT, we chose to increase the alpha power of the T3 channel and reduce that of the T4 channel. Compared with the SMR group, which increased the SMR power on the three channels at the same time, the signal-to-noise ratio (SNR) obtained by alpha training may be lower. Therefore, the experimental results that alpha training is more difficult than SMR training may also be affected by the SNR of the training paradigm. Subsequent research should take the SNR of the feedback signal and the difficulty of feedback training into consideration.

## Conclusion

To compare the efficacy, trainability and neuroplasticity effects of SMR and alpha SP-NFT, 45 participants were recruited into the experiment, and 30 participants trained with SP-NFT during six sessions in 3 weeks, respectively. Through the analysis of the experimental results, the following main conclusions were obtained:

(1) By comparing the results before and after SP-NFT, we showed that the shooting performance of the SMR group after SP-NFT was significantly higher than that before SP-NFT, while the shooting performance of the Alpha group after SP-NFT was lower than that before SP-NFT. Thus, for non-professional shooters, enhanced SMR may be more effective to improve shooting performance than enhancing the alpha power difference in the left and right temporal regions.
(2) The results regarding neurofeedback trainability scores and feedback features show that the participants can master the SP-NFT technology correctly to improve the neurofeedback trainability scores and feedback features through training. By comparing the subjective task appraisal results, we found that SMR NFT was subjectively assessed as less difficult and was easier to master than alpha NFT.
(3) A comparison of the results of EEG before and after SP-NFT showed that the power spectrum of COI and that of the whole brain of the participants presented direct and specific changes, indicating that SP-NFT may promote neuroplasticity and has a continuous effect on resting EEG activity.

Overall, the results in this article provide some evidence of the effects of SP-NFT on trainability and neuroplasticity and further contribute to the application of SP-NFT to improve shooting performance stage.

## Data Availability Statement

The datasets generated for this study are available on request to the corresponding author.

## Ethics Statement

The studies involving human participants were reviewed and approved by Engineering University of the Chinese People’s Armed Police Force ethics committee. The patients/participants provided their written informed consent to participate in this study.

## Author Contributions

AG collected experimental data and wrote the original manuscript. WN analyzed experiment results. CJ and YF designed experiments and revised manuscript. EY provided important advice and help on key content of the manuscript.

## Conflict of Interest

The authors declare that the research was conducted in the absence of any commercial or financial relationships that could be construed as a potential conflict of interest.
